# Immunizing Adult Female Mice with a TcpA-A2-CTB Chimera Provides a High Level of Protection for Their Pups in the Infant Mouse Model of Cholera

**DOI:** 10.1371/journal.pntd.0003356

**Published:** 2014-12-04

**Authors:** Gregory A. Price, Randall K. Holmes

**Affiliations:** Department of Immunology and Microbiology, University of Colorado School of Medicine, Aurora, Colorado, United States of America; Massachusetts General Hospital, United States of America

## Abstract

*Vibrio cholerae* expresses two primary virulence factors, cholera toxin (CT) and the toxin-coregulated pilus (TCP). CT causes profuse watery diarrhea, and TCP (composed of repeating copies of the major pilin TcpA) is required for intestinal colonization by *V. cholerae*. Antibodies to CT or TcpA can protect against cholera in animal models. We developed a TcpA holotoxin-like chimera (TcpA-A2-CTB) to elicit both anti-TcpA and anti-CTB antibodies and evaluated its immunogenicity and protective efficacy in the infant mouse model of cholera. Adult female CD-1 mice were immunized intraperitoneally three times with the TcpA-A2-CTB chimera and compared with similar groups immunized with a TcpA+CTB mixture, TcpA alone, TcpA with *Salmonella typhimurium* flagellin subunit FliC as adjuvant, or CTB alone. Blood and fecal samples were analyzed for antigen-specific IgG or IgA, respectively, using quantitative ELISA. Immunized females were mated; their reared offspring were challenged orogastrically with 10 or 20 LD_50_ of *V. cholerae* El Tor N16961; and vaccine efficacy was assessed by survival of the challenged pups at 48 hrs. All pups from dams immunized with the TcpA-A2-CTB chimera or the TcpA+CTB mixture survived at both challenge doses. In contrast, no pups from dams immunized with TcpA+FliC or CTB alone survived at the 20 LD_50_ challenge dose, although the anti-TcpA or anti-CTB antibody level elicited by these immunizations was comparable to the corresponding antibody level achieved by immunization with TcpA-A2-CTB or TcpA+CTB. Taken together, these findings comprise strong preliminary evidence for synergistic action between anti-TcpA and anti-CTB antibodies in protecting mice against cholera. Weight loss analysis showed that only immunization of dams with TcpA-A2-CTB chimera or TcpA+CTB mixture protected their pups against excess weight loss from severe diarrhea. These data support the concept of including both TcpA and CTB as immunogens in development of an effective multivalent subunit vaccine against *V. cholerae*.

## Author Summary

*Vibrio cholerae* is the bacterium that causes cholera, a pandemic diarrheal disease transmitted by ingestion of contaminated food or water. We developed a novel vaccine containing two protective antigens of *V. cholerae*, TcpA and CTB, incorporated into a defined oligomeric protein chimera. CTB is the non-toxic binding domain of cholera toxin, the protein that causes profuse watery diarrhea in cholera patients. TcpA is the subunit of the toxin-coregulated pilus, a *V. cholerae* surface structure that is required for intestinal colonization and disease. Intraperitoneal immunization of adult female mice with this TcpA-A2-CTB chimera elicited stronger early anti-TcpA responses and equivalent anti-CTB responses compared to immunizing with a TcpA+CTB mixture. Furthermore, all reared infant mice from females immunized with the chimera or TcpA+CTB were protected against a large challenge dose of *V. cholerae* that was sufficient to kill all infant mice from non-immunized control and TcpA- or CTB-immunized adults. Our study supports the concept of including both TcpA and CTB as antigens in development of a safe and effective subunit vaccine against cholera.

## Introduction

Cholera is an intestinal infection that is associated with acute watery diarrhea and is caused by the Gram-negative bacillus *Vibrio cholerae*. Cholera is spread by the ingestion of contaminated food and water. An estimated 3–5 million people are infected yearly with cholera, resulting in approximately 100,000 deaths [1]. Cholera is endemic in over 50 countries in the developing world where risk factors such as over-crowding, lack of clean food and water, and poor sanitation allow for its persistence in the environment [1], [2]. Cholera can cause severe life-threatening dehydration, and stool outputs as high as 500–1000 ml/hr can rapidly lead to death in untreated patients [2]. The most effective treatment for cholera is rehydration therapy, and if treatment is started early enough the case fatality rate (CFR) is below 1% [3]. However, it is often difficult for poor and impoverished patients to have access to medical treatment. Cholera can be prevented by vaccination. In 2011, a review of published studies on five variants of an oral whole-cell killed (WCK) cholera vaccine showed that their overall protective efficacy after two years was 62% in adults, they were less effective in children under 5 years of age, and they were unlikely to provide protection beyond three years [4]. In 2013, a study of a re-formulated WCK oral cholera vaccine in Kolkata, India, showed a 5-year cumulative protective efficacy of 65% in all individuals over 1 year of age, but a lower 5-year protective efficacy of 42% in children from 1 to 5 years of age [5]. Nevertheless, a recent critical analysis concludes that current WCK cholera vaccines are poorly suited to control endemic or epidemic cholera because of limited efficacy in young children, requirements for multiple doses, a cold chain, and complex delivery logistics, and costs that are high for resource-poor regions [6]. Finding solutions for such issues is an important goal for developing improved cholera vaccines.

We are investigating development of safe and effective subunit vaccines against cholera. Subunit vaccines can present important virulence determinants such as colonization factors and toxins that might not be present or highly immunogenic in a WCK or living attenuated vaccine. For *Vibrio cholerae*, two essential virulence factors are cholera toxin (CT) and the toxin-coregulated pilus (TCP). CT is an AB_5_ toxin that is primarily responsible for diarrhea in cholera. CT consists of a monomeric A subunit (CTA) and a homopentameric B subunit (CTB) [7], binds to monosialosyl ganglioside G_M1_ receptors on enterocytes [8], enters them by endocytosis, trafficks to the endoplasmic reticulum, and releases its CTA1 fragment for retrotranslocation into the cytosol [9]. In the cytosol, CTA1 ADP ribosylates the α subunit of Gs (Gsα), resulting in activation of adenylate cyclase, accumulation of intracellular adenosine-3,5-cyclic monophosphate (cAMP), and downstream events including an efflux of ions and water into the intestinal lumen that presents clinically as diarrhea [10], [11].

TCP is a type IV pilus composed of repeating subunits of the major pilin subunit TcpA [12]. TCP functions *in vivo* by mediating bacterium-bacterium interactions that are essential for the formation of microcolonies on the surface of enterocytes in the small intestine [13], [14]. In recent infant mouse experiments, TCP has also been demonstrated to mediate attachment of *V. cholerae* to epithelial cells and to form TCP matrices that engulf the bacteria and may help to protect them from antimicrobial agents [14].

The importance of CT and TCP for *V. cholerae* virulence has been demonstrated both in animals and in humans, as strains of *V. cholerae* that fail to produce either CT or TcpA are severely attenuated [12], [15]–[17]. Immunization with CT or non-toxic derivatives of CT has been shown to elicit protective immunity in animal models but not in humans [18]–[21]. Passive orogastric administration of anti-TCP antibodies can provide excellent protection in the infant mouse model of cholera [22], [23], but immunization of humans with intact TCP or with TcpA subunits has not yet been investigated.

In the study reported here, we tested recombinant forms of TcpA and CTB (either alone, in combination, or as a holotoxin-like chimera) as candidate cholera vaccines in the infant mouse model of cholera.

## Materials and Methods

### Ethics statement

All procedures involving experimental animals were approved by the University of Colorado Denver (UCD) Animal Care and Use Committee. These procedures were done in compliance with all institutional and governmental requirements and regulations regarding the appropriate ethical use of animals in research. UCD is accredited by the Association for the Assessment and Accreditation of Laboratory Animal Care, International (file number 00235).

### Construction of expression plasmids

**Figure 1:**
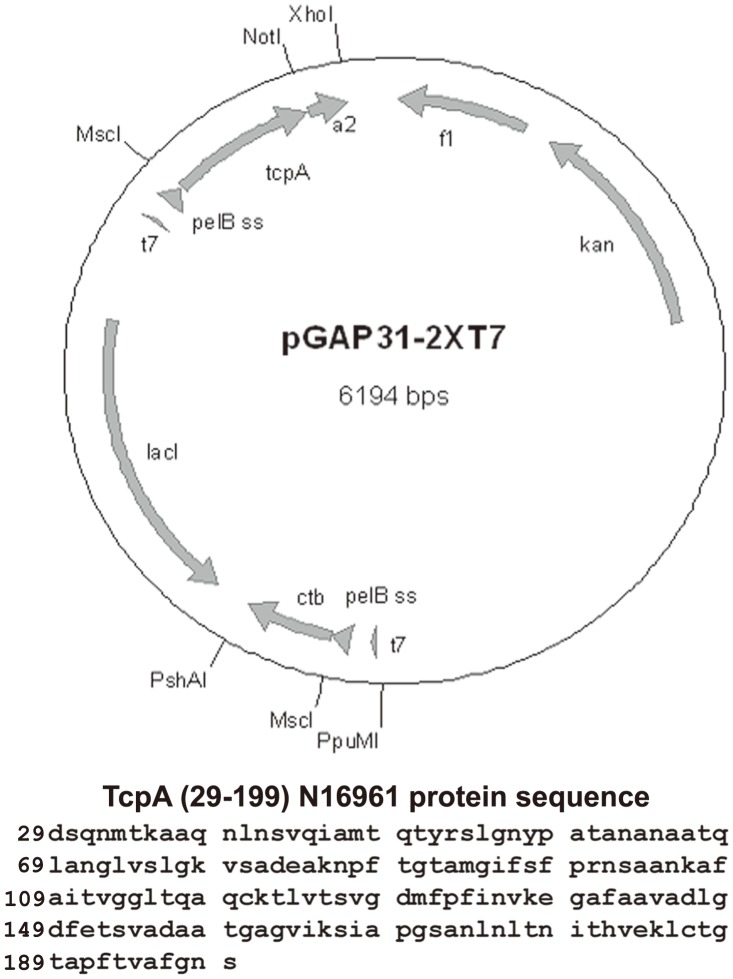
Schematic representation of the dual T7 promoter TcpA-A2-CTB expression plasmid pGAP31-2XT7 and amino acid sequence of the TcpA (29–199) fragment encoded by *V.cholerae* El Tor N16961. Gene fragment *tcpA* (encoding residues 29–199 of mature TcpA) and gene fragment *a2* (encoding the A2 polypeptide corresponding to residues 196–240 of mature CTA) were cloned in-frame and downstream of gene fragment *pelB* (encoding the PelB signal sequence), and the recombinant gene was expressed under control of an IPTG-inducible T7 promoter. Gene fragment *ctb* (encoding mature CTB) was cloned in-frame and downstream of another *pelB* gene fragment, and the recombinant gene was expressed under control of a second IPTG-inducible T7 promoter. The PelB signal sequences directed the TcpA-A2 fusion protein and CTB into the periplasm in *E. coli*, and the TcpA-A2-CTB chimera was assembled spontaneously in the periplasm from one TcpA-A2 polypeptide and five CTB polypeptides. The amino acid sequence of the TcpA fragment (residues 29–199) that is included in the TcpA-A2 fusion protein is shown at the bottom of the figure.

All genes were PCR amplified using genomic DNA from *V. cholerae* El Tor strain N16961 and for FliC from genomic DNA from *Salmonella typhimurium* strain 14028s. The TcpA-A2-CTB chimera dual promoter expression plasmid pGAP31-2XT7 was constructed in several steps. First, the *a2* gene fragment encoding CTA2 was amplified by PCR using the forward primer oA2-Fnot and the reverse primer oA2-Rxho containing the NotI and XhoI restriction sites respectively (Table 1; restriction sites shown in bold). The primer oA2-Fnot contained a point mutation in the *a2* coding sequence to change a cysteine to a serine (Table 1; point mutation underlined). Second, the *tcpA* gene fragment encoding residues 29–199 of the mature TcpA polypeptide was PCR amplified using the forward primer oTcpAn16961-Fmsc and the reverse primer oTcpAn16961-Rnot containing the MscI and NotI restriction sites respectively (Table 1). Previous studies demonstrated this polypeptide to be soluble, surface-exposed, and immunogenic [24], [25]. Third, the *a2* and *tcpA* genes were subcloned into an altered pET22b(+)[EMB Biosciences, Gibbstown, NJ] expression plasmid in which the ampicillin resistance marker was replaced with a kanamycin resistance marker. The kanamycin resistance marker was obtained from pET28b(+) [EMD Biosciences, Gibbstown, NJ] which was cut with EcoRI and PpuMI, and the isolated restriction fragment was then ligated into pET22b(+). The insertion of the *tcpA* and *a2* gene fragments was downstream and in frame with the *pelB* signal sequence. Fourth, a second T7 promoter containing the mature *ctb* gene in frame with the *pelB* signal sequence was PCR amplified from the expression plasmid pGAP20K [26] using the forward primer oT7-FppuMI and the reverse primer oCTB-RpshAI. Finally, the *t7-pelB-ctb* gene product was subcloned into the PshAI and PpuMI sites of the TcpA-A2 expression plasmid generated in step 3 above, thereby creating the dual T7 promoter expression plasmid pGAP31-2XT7 (Fig. 1).

**Table 1:** Primer sequences.

Primer	Sequence
oTcpAn16961-Fmsc	GC**TGGCCA**CAGATTCGCAGAATATGACTAAG
oTcpAn16961-Rnot	T**GCGGCCGC**ACTGTTACCAAAAGCTACTG
oA2-Fnot	T**GCGGCCGC**AAGTAATACTAGCGATGAAAA
oA2-Rxho	T**CTCGAG**TCATAATTCATCCTTAAT
oT7-FppuMI	T**GGGTCCT**AGATCTCGATCCCGCGAAAT
oCTB-RpshAI	CT**GACTATCGTC**TTAATTTGCCATACTAATTG
oCTB-Fmsc	CGC**TGGCCA**CACCTCAAAATATTACTG
oCTB-Rxho	TTT**CTCGAG**TTAATTTGCCATACTAATTGC
oTcpAn16961-Fnde	A**CATATG**GATTCGCAGAATATGACTAAG
oTcpAn16961-Rxho	T**CTCGAG**TTAACTGTTACCAAAAGCTA
oFliC-Fnde	T**CATATG**GCACAAGTCATTAATAC
oFliC-Rxho	T**CTCGAG**TTAACGCAGTAAAGAGAGGAC

The CTB expression plasmid pGAP20K, which encodes the *ctxB* allele from *V. cholerae* El Tor strain N16961, was constructed as previously described [26]. The N-terminal 6-histidine tagged-TcpA expression plasmid was created by PCR amplifying the *tcpA* gene fragment encoding residues 29–199 of the mature TcpA polypeptide (Fig. 1) using the forward primer oTcpAn16961-Fnde and the reverse primer oTcpAn16961-Rxho (Table 1). This was inserted into pET28b(+) using the NdeI and XhoI restriction sites, downstream and in frame of an N-terminal 6-his tag, creating the expression plasmid pGAP33.

The N-terminal 6-histidine-tagged-FliC expression plasmid was created by PCR amplifying the Salmonella typhimurium *fliC* gene using the forward primer oFliC-Fnde and the reverse primer oFliC-Rxho (Table 1). This was inserted into pET28b(+) using the NdeI and XhoI restriction sites, downstream and in frame with the N-terminal 6-his tag, creating the expression plasmid pGAP32.

### Recombinant protein production and purification

The TcpA-A2-CTB chimera was produced in *Escherichia coli* BL-21(DE3) Star™ cells (Invitrogen, Grand Island, NY). Half-liter cultures were grown in NZTCYM medium pH 7.5 (1% N-Z-amine AS [Sigma, St. Louis, MO], tryptone 1%, NaCl 0.5%, yeast extract 0.5%, casamino acids 0.1%, MgSO4 0.2%) and 100 µg/ml kanamycin at 37°C, 250 rpm until cultures reached an OD_600_ of ∼3.0. The cultures were then placed at 16°C and 250 rpm for 30 minutes to acclimate to the new temperature then induced with 0.2 mM IPTG and grown overnight at 16°C for ∼16–18 hrs. 6His-TcpA(29–199) was produced in SHuffle™ T7 Express *E. coli* (NEB, Ipswich, MA) in half liter cultures of TCYM media pH 7.5 (tryptone 1%, NaCl 0.5%, yeast extract 0.5%, casamino acids 0.1%, MgSO4 0.2%) and 100 µg/ml kanamycin at 37°C, 250 rpm until cultures reached an OD_600_ of ∼3.0. The cultures were then placed at 16°C, 250 rpm for 30 minutes, and then induced with 0.1 mM IPTG and grown overnight as above. Cultures of *E. coli* BL-21(DE3) cells producing 6His-FliC were grown in half liter cultures of TCYM pH 7.5 at 37°C 250 rpm until they reached an OD_600_ of ∼2.0–3.0. After acclimating to 30°C with shaking at 250 rpm for 30 minutes, the cultures were then induced with 0.5 mM IPTG and incubated for 4 hrs. CTB was grown and induced as described previously [26].

Preparation of the bacterial extracts and primary metal affinity purification of all proteins was performed as described previously [26]. A secondary purification step on the strong cation-exchange resin POROS 20 HS (Applied Biosystems, Carlsbad, CA) was performed for TcpA-A2-CTB, 6His-TcpA(29–199), and CTB. Both the TcpA-A2-CTB chimera and 6His-TcpA(29–199) were purified under the same conditions. Each was dialyzed overnight at 4°C in 25 mM potassium phosphate buffer pH 6.8. Soluble and filtered protein was loaded onto a POROS 20 HS (Applied Biosystems, Carlsbad, CA) column, and eluted with a linear 0 to 0.5 M gradient of NaCl in 25 mM potassium phosphate buffer at pH 6.8. This purified, soluble, recombinant 6His-TcpA(29–199) protein is subsequently called TcpA.

For CTB, the protein was first desalted using Zeba™ Desalt Spin Columns (Thermo Fisher Scientific, Rockford, IL) following the manufacturer's protocol. CTB was desalted into potassium phosphate buffer pH 6.6 and then filtered through a 0.45 µM syringe filter to remove precipitated material. An ion-exchange purification step was then conducted using POROS 20 HS (Applied Biosystems, Carlsbad, CA) resin. The bound protein was eluted using a linear 0 to 0.5 M gradient of NaCl in 25 mM potassium phosphate buffer pH 6.6.

6His-FliC was dialyzed overnight against 20 mM Tris-Cl pH 8.0. An ion-exchange purification step was then conducted using the strong anion-exchange resin POROS 20 HQ (Applied Biosystems, Carlsbad, CA). The bound protein was eluted from the resin using a linear gradient of 0 to 0.5 M NaCl in 20 mM Tris-Cl buffer at pH 8.0. This purified, soluble, recombinant 6His-FliC protein is subsequently called FliC. Following purification TcpA, CTB, and FliC were dialyzed overnight at 4°C against 1× PBS and stored at −80°C. The TcpA-A2-CTB chimera was dialyzed overnight at 4°C against 50 mM Tris buffer containing 200 mM NaCl and 1 mM EDTA at pH 7.5.

### Immunization and sample collection

Female CD-1 mice, 6–8 weeks old, were purchased from Charles River Labs and given food and water ad libitum. Groups of 7–10 mice were immunized three times IP at 14 day intervals. The group that was immunized with the TcpA-A2-CTB chimera received 50 µg/dose. All other groups received amounts of each antigen that were equimolar with the amount of the corresponding antigenic component in a 50-µg dose of the chimera. For the groups immunized with TcpA combined with FliC or FliC alone, the dose of FliC administered for the first or second immunization was 5 µg, and a 2 µg dose was administered for the final immunization. Blood and fecal samples were collected one day prior to the initial immunization (Day −1) and on days 21 and 42. Sample collection and processing was performed as previously described [26]. To obtain blood samples from infant mice, the pups were first asphyxiated with CO_2_, and then a scalpel was used to sever the cervical spinal cord. A heparinized capillary tube was used to collect blood that seeped from the incision. One or two pups were used per dam, and blood was collected on the same day that siblings were challenged with *V. cholerae*. Blood was pooled if two siblings were used to obtain sera.

### Quantitative and G_M1_ ganglioside ELISAs

To measure antigen-specific antibody amounts in serum and fecal extracts, we used quantitative ELISAs as described previously [26]. The concentration or amount of antigen-specific IgG or IgA antibodies in unknown samples was determined by interpolation from a standard curve using KC4 v3.4 software (Bio-Tek. Winooski, VT). G_M1_ ganglioside ELISAs were performed as previously described [26] using sera from rabbits immunized with recombinant TcpA or CTB.

### Infant mouse challenges

The infant mouse challenges were performed as previously described [21], [26]. All pups were six days old at the time of inoculation. The pups were monitored for survival over the course of 48 hrs. Pup weights were recorded immediately prior to inoculation and at 24 and 48 hours post-infection. For pups that died prior to 24 hours, their carcass weights were measured and included with the group at 24 hours. For pups that died between 24 and 48 hours, their carcass weights were measured and included with the group at 48-hours.

### Statistical analyses

All statistical comparisons were performed using GraphPad PRISM 4 (La Jolla, CA). ANOVA analysis using the Tukey-Kramer post-test was used to determine statistical significance between immunization groups for antigen-specific antibody concentration differences and for weight loss differences between immunization groups for the infant mouse challenge. Within-group statistical differences for antigen-specific antibody amounts at days 21 and 42 were analyzed using a paired two-tailed t-test. Survival curves were generated using Kaplan-Meier method, and statistical differences between experimental groups were determined using the log-rank (Mantel-Cox) test. *P* values less than 0.05 were considered significant.

### Accession numbers

Both gene and protein sequences for the antigens used in this study can be found in the National Center for Biotechnology Information (NCBI) database. The genes (accession numbers) are as follows: *ctxB* (NC_002505), *tcpA* (AF536868), and *fliC* (NC_016856). The protein sequences (accession numbers) are as follows: CTB (NP_231099), TcpA (AAN15109) and FliC (YP_005237927).

## Results

### Analysis of the TcpA-A2-CTB chimera

**Figure 2:**
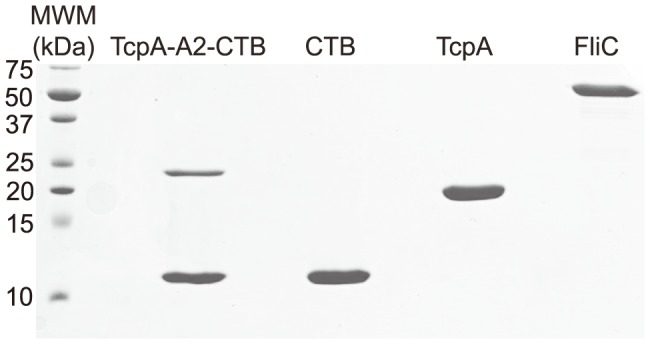
Analysis of purified TcpA-A2-CTB chimera and TcpA, CTB, and FliC proteins by SDS-PAGE. All samples were reduced and boiled prior to loading and electrophoresis on a 15% SDS-PAGE gel that was subsequently stained with Coomassie blue dye. Denaturation and heat treatment caused the TcpA-A2-CTB chimera to separate into the TcpA-A2 fusion protein (∼23 kDa) and CTB monomers (∼11.5 kDa). The pentameric CTB also separated into CTB monomers (∼11.5 kDa). TcpA and FliC migrated as single polypeptides of ∼20 kDa and ∼54 kDa respectively. The left lane shows a ladder of molecular weight markers (MWM) with molecular masses (kDa) of specific markers indicated by numbers at the left side of the gel.

**Figure 3:**
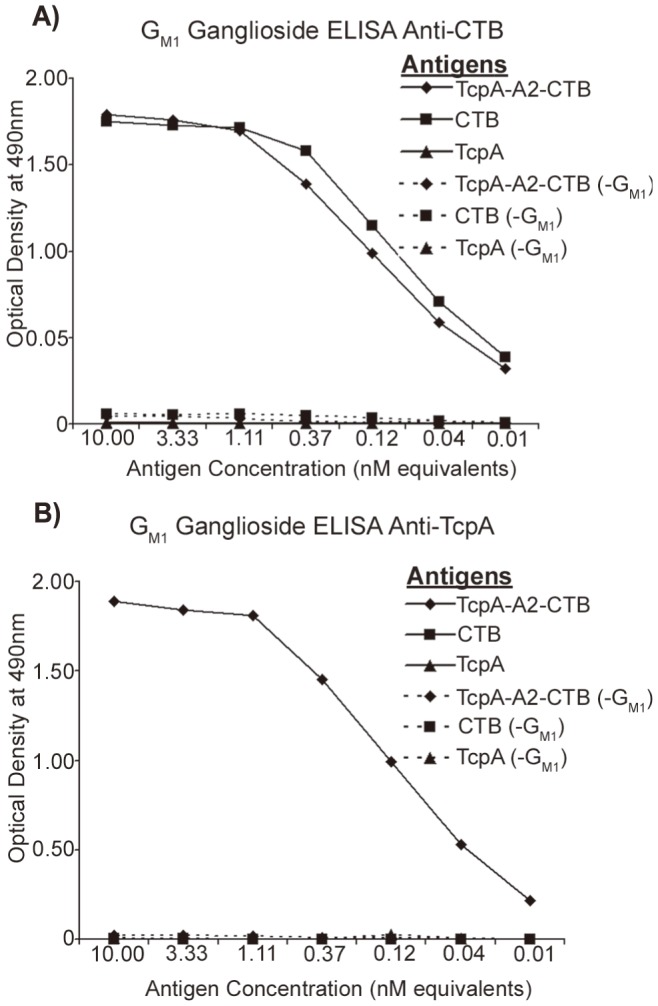
Analysis of binding activity and immunoreactivity of the TcpA-A2-CTB chimera and the CTB and TcpA proteins by ganglioside G_M1_ ELISA assays. Equimolar amounts of each protein were loaded and serially diluted into wells of the ganglioside G_M1_-coated ELISA plates or control plates without bound ganglioside G_M1_ (-G_M1_). After incubation and washing, the plates were probed with either rabbit anti-CTB antiserum (A) or anti-TcpA antiserum (B), followed by secondary probing with HRP-conjugated goat anti-rabbit IgG. Plates were read at OD_490_ and the optical densities recorded at each dilution. The TcpA-A2-CTB chimera and CTB protein, but not TcpA, exhibited specific binding to immobilized ganglioside G_M1_ on the plates. The bound Tcp-A2-CTB chimera and bound CTB were both immunoreactive with anti-CTB (A), but only the bound Tcp-A2-CTB chimera was immunoreactive with anti-TcpA (B).

The TcpA-A2-CTB chimera was expressed in *E. coli* using the dual T7 promoter plasmid pGAP31-2XT7 (Fig. 1), and it was purified using sequential metal affinity chromatography and ion-exchange chromatography. Upon heating and denaturation, the purified chimera separated into the TcpA-A2 fusion protein (∼23 kDa) and monomeric CTB (∼11.5 kDa; Fig. 2). TcpA-A2 migrated more slowly than TcpA, reflecting the greater molecular mass of the fusion protein due to the presence of CTA2 at its carboxyl-terminus (Fig. 2). We used G_M1_ ganglioside ELISAs to demonstrate immunoreactivity of the TcpA-A2 moiety and both immunoreactivity and ganglioside G_M1_ receptor-binding activity of the pentameric CTB moiety of the purified TcpA-A2-CTB chimera (Fig. 3). Solutions containing equimolar amounts of the TcpA-A2-CTB chimera, or TcpA alone, or CTB alone, were serially diluted and added to ELISA plates that had previously been coated with ganglioside G_M1_ and then blocked to prevent nonspecific binding of the test antigens. Subsequently the plates were washed and then probed with either anti-CTB rabbit antiserum or anti-TcpA rabbit antiserum. The TcpA-A2-CTB chimera and CTB (but not TcpA) bound avidly to the plates coated with G_M1_ ganglioside. Bound TcpA-A2-CTB chimera and bound CTB were both detected with anti-CTB antiserum (Fig. 3, top), but only the bound chimera was detected with anti-TcpA antiserum (Fig. 3, bottom). None of these antigens bound above background levels to control plates that were blocked but not coated with ganglioside G_M1_. Taken together, these results showed that the TcpA-A2-CTB chimera is a bi-functional oligomeric complex that exhibits TcpA immunoreactivity associated with its TcpA-A2 fusion polypeptide and both CTB immunoreactivity and ganglioside G_M1_ binding activity associated with its pentameric CTB subunit.

### Antibody responses following IP immunization

**Figure 4:**
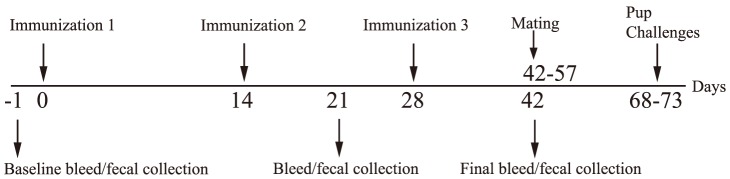
Mouse procedures timeline. Female CD-1 mice were immunized 3 times at 14-day intervals by the IP route starting at day 0. Blood and fecal samples were collected on days −1, 21, and 42. Following the final bleed, the females were mated 1-to-1 with 12-week old male CD-1 mice. The females were monitored for birth and 6-day old pups were used for challenge with live *V. cholerae* N16961.

**Figure 5:**
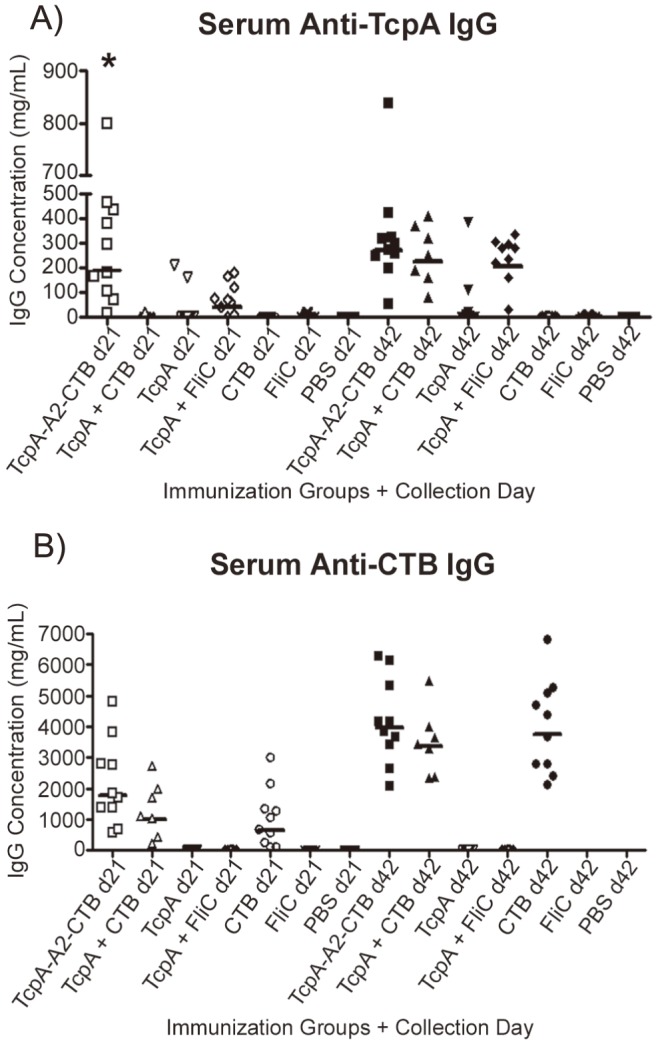
Concentrations of anti-TcpA and anti-CTB IgG antibodies in sera from adult mice after IP immunization with TcpA-A2-CTB chimera, TcpA+CTB, TcpA, TcpA+FliC, CTB, FliC, or PBS. The concentrations of anti-TcpA (A) and anti-CTB (B) antibodies were measured by quantitative ELISA in serum samples collected on days 21 and 42 from the mice in each immunization group. Each data point represents the concentration of the indicated antigen-specific IgG antibody in serum from an individual mouse, and each horizontal bar represents the geometric mean value of the indicated antigen-specific IgG antibody concentrations for the corresponding immunization group. Statistical differences between groups were evaluated using ANOVA with the Tukey-Kramer post-test analysis (* indicates *P*<0.001 versus all other groups at day 21).

To compare the immunogenicity of the TcpA-A2-CTB chimera with non-chimeric forms of TcpA and CTB, we immunized groups of 7 to 10 female CD-1 mice three times by the IP route according to the immunization timeline shown in figure 4. In an attempt to show whether inherent immunogenicity of recombinant TcpA protein could be enhanced by use of an adjuvant, we included separate groups of mice immunized either with TcpA alone or with TcpA plus the recombinant flagellin subunit protein FliC from Salmonella typhimurium. FliC is a toll receptor 5 (TLR5) agonist and has been demonstrated previously to act as an adjuvant for co-administered antigens [27]. Serum and fecal antigen-specific antibody responses were measured using quantitative ELISA for the samples collected on days 21 and 42 (Fig. 4). We found that immunization with the TcpA-A2-CTB chimera elicited a significantly higher mean concentration of serum anti-TcpA IgG on day 21 compared with all other immunization groups (P<0.001, Fig. 5A). However by day 42, 14 days following the third and final immunization, the mean serum anti-TcpA IgG concentrations for the groups immunized with the TcpA-A2-CTB chimera, the TcpA+CTB mixture, and the TcpA+FliC mixture were comparable (P>0.05), but all were significantly greater than the mean serum anti-TcpA IgG concentration after immunization with TcpA alone (P<0.01). These results demonstrated that either incorporating the TcpA-A2 fusion protein into the TcpA-A2-CTB chimera or administering TcpA together with CTB or FliC enhanced the immunogenicity of TcpA, and the results for the samples collected at day 21 suggest that the TcpA-A2-CTB chimera presented the TcpA moiety in its most immunogenic form. Serum anti-CTB IgG responses were robust in all groups that received CTB, either as CTB alone, as TcpA+CTB, or as the TcpA-A2-CTB chimera (Fig. 5B). There were no significant differences in mean anti-CTB IgG concentrations between these groups either at day 21 (P>0.05) or at day 42 (P>0.05), although the mean titers were significantly higher at day 42 than at day 21 (P<0.0001). As expected, control mice immunized with PBS did not develop any detectable anti-TcpA or anti-CTB antibodies.

**Figure 6:**
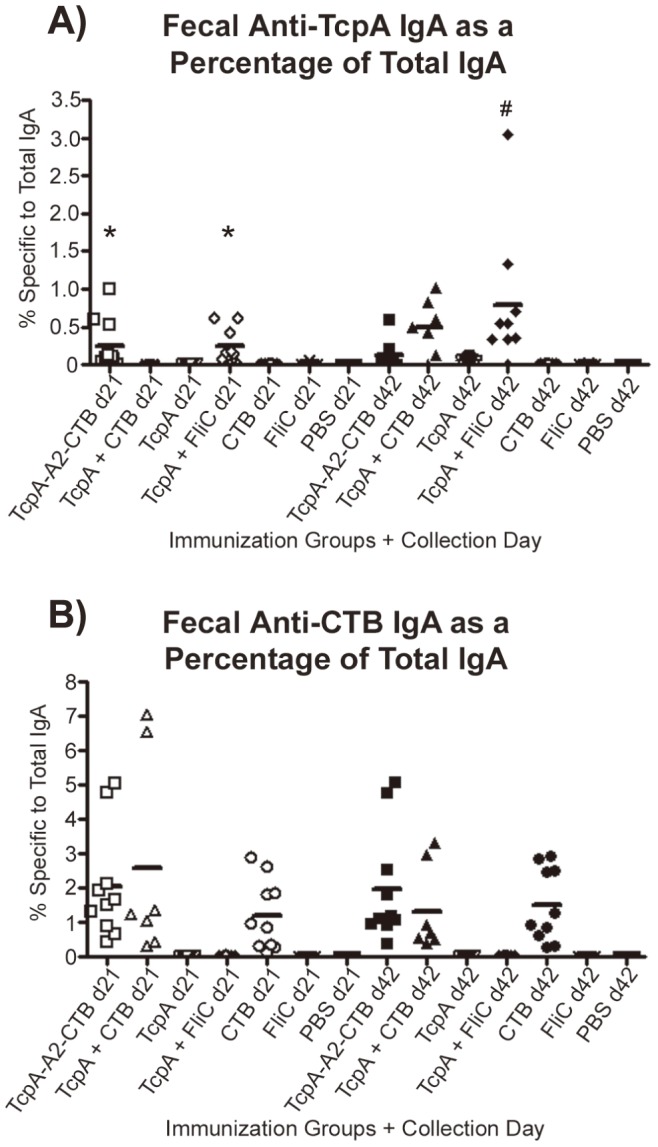
Measurements of anti-TcpA and anti-CTB IgA antibodies in fecal extracts from adult mice after immunization with TcpA-A2-CTB chimera, TcpA+CTB, TcpA, TcpA+FliC, CTB, FliC, or PBS. Fecal pellets were collected from each mouse on days 21 and 42 for each immunization group, and fecal extracts were prepared. The amount of anti-TcpA IgA antibody (A) or anti-CTB IgA antibody (B) in each fecal extract was determined by ELISA and expressed as a fraction of the total IgA immunoglobulin in that extract. Each point represents data from one mouse, and each horizontal line represents the geometric mean value for the corresponding group. Statistical differences between groups were evaluated using ANOVA with the Tukey-Kramer post-test analysis (* indicates groups with P<0.05 versus all other d21 immunization groups not marked by *; ^#^ indicates P<0.05 versus all other d42 immunization groups except for the TcpA+CTB group).

The amounts of antigen-specific IgA antibody and total IgA immunoglobulin were measured in each fecal extract. In figure 6, the amount of antigen-specific IgA antibody is shown as a percentage of total IgA for each fecal extract. We normalized the data in this way to minimize differences that might result from mouse-to-mouse variations in production of fecal IgA or sample-to-sample variations in recovery of IgA from the fecal specimens. Immunization with either the TcpA-A2-CTB chimera or the TcpA+FliC mixture elicited a significantly greater mean fecal anti-TcpA IgA response on day 21 than immunization with any of the other antigens (Fig. 6A, P<0.05). Interestingly, at day 42 the mean fecal anti-TcpA IgA response to immunization with the TcpA-A2-CTB chimera was less than at day 21, but the mean fecal anti-TcpA IgA response to immunization with TcpA+FliC was greater than at day 21. However, neither of these pairwise differences in mean antibody amounts between days 21 and 42 was significant (P>0.05). On day 42 the mean fecal anti-TcpA IgA response to immunization with TcpA+CTB had increased dramatically to a level that was comparable to the TcpA+FliC immunization group (P>0.05), and both were significantly greater than mean values for all other immunization groups at day 42 (P<0.05) (Fig. 6A).

As with the serum CTB-specific IgG responses, the fecal anti-CTB IgA responses at day 42 were not significantly different in any of the groups that received CTB as an immunogen, either as CTB alone, as TcpA+CTB, or as the TcpA-A2-CTB chimera (Fig. 6B; *P*>0.05). In contrast to the increases in the serum CTB IgG concentrations that occurred from day 21 to day 42, however, the fecal CTB-specific IgA percentages on day 21 and day 42 were comparable (P>0.05). Finally, for each group that was immunized both with TcpA and CTB (e.g., immunized with either TcpA-A2-CTB or TcpA+CTB), the mean percentage of fecal anti-CTB IgA was greater than the mean percentage of fecal anti-TcpA IgA in the same group, both at day 21 and at day 42 (compare results and note differences in scales for the Y axes for Figs. 6A and 6B).

### Analysis of survival in the suckling mouse model of cholera

**Figure 7:**
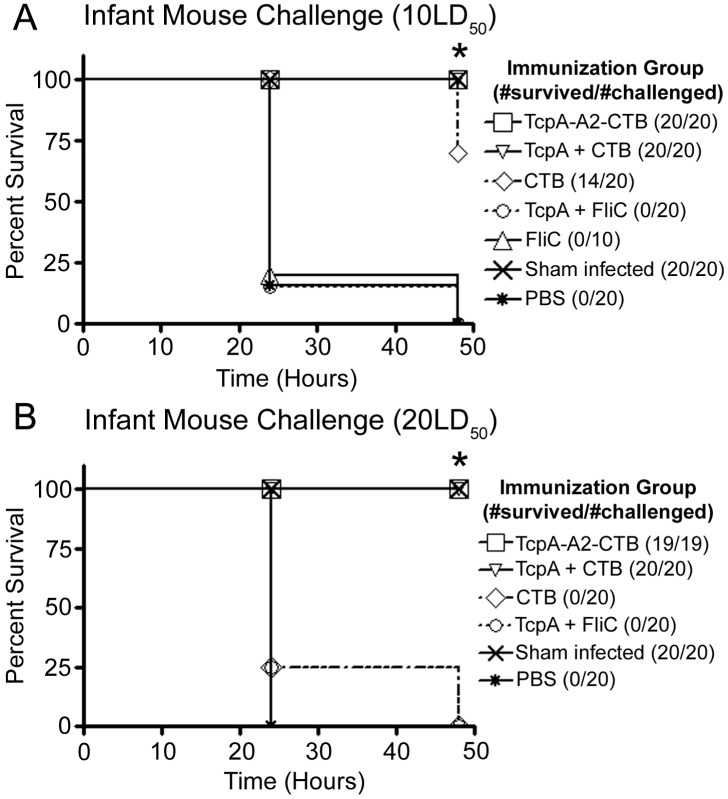
Kaplan-Meier survival curves for mouse pups born to immunized or non-immunized dams and challenged with 10 or 20 LD50 of *V. cholerae* El Tor strain N16961. Six-day old mouse pups were challenged with live *V. cholerae* at a dose of 10 LD_50_ (A) or 20 LD_50_ (B) and monitored for survival for 48 hrs. Statistical differences between groups were evaluated using the log-rank (Mantel-Cox) test. In 7A, * indicates that survival of the groups immunized with TcpA-A2-CTB or TcpA+CTB differed significantly both from the group immunized with CTB (P = 0.0087) and from the groups immunized with TcpA+FliC, FliC, or PBS (P<0.0001). In 7B, * indicates that survival of the groups immunized with TcpA-A2-CTB or TcpA+CTB differed significantly from the groups immunized with CTB, TcpA+FliC, or PBS (P<0.0001). The group immunized only with FliC was not challenged with *V. cholerae* at the 20 LD_50_ dose.

To compare the protective efficacies of selected vaccine regimens, immunized dams were mated (see timeline in Fig. 4) and groups of their reared 6-day old pups were challenged orogastrically with 10 LD_50_ of *V. cholerae* El Tor strain N16961 and monitored for survival at 24 and 48 hrs (Fig. 7A). All pups from dams immunized either with TcpA-A2-CTB chimera (n = 20) or TcpA+CTB (n = 20), and all sham-infected pups (n = 20) survived for 48 hrs. All pups from dams immunized with CTB alone (n = 20) survived for 24 hrs, and 70% survived for 48 hrs (*P* = 0.0087 *vs.* pups immunized with TcpA-A2-CTB or TcpA+CTB). In contrast, pups from dams immunized with TcpA+FliC (n = 20) or FliC alone (n = 10), like pups from PBS immunized dams (n = 20), experienced 77.5–80% mortality by 24 hrs and 100% mortality by 48 hrs (*P*<0.0001 *vs.* pups immunized with TcpA-A2-CTB or TcpA+CTB). We did not challenge pups from dams immunized with TcpA alone, because those dams were previously shown to have much lower serum and fecal anti-TcpA antibody levels than dams immunized with TcpA+FliC (Figs. 5A and 6A).

To investigate under more stringent conditions the contributions of anti-TcpA and anti-CTB antibodies in protecting infant mice against cholera, we challenged additional pups from the immunized dams with a higher 20 LD_50_ challenge dose of *V. cholerae* El Tor strain N16961 (Fig. 7B). All pups from dams immunized with TcpA-A2-CTB chimera (n = 19) or TcpA+CTB (n = 20), and all sham-infected pups (n = 20), survived for 48 hrs. In contrast, no pups from dams immunized with CTB alone (n = 20), TcpA+FliC (n = 20), or PBS (n = 20), survived for 48 hrs (*P*<0.001 vs each of the three previous groups). The mean concentrations of serum anti-TcpA IgG at day 42 did not differ significantly in dams immunized with TcpA-A2-CTB chimera, TcpA+CTB, or TcpA+FliC (Fig. 5A, P>0.05), and the mean percentages of fecal anti-TcpA IgA also did not differ significantly in the dams immunized with TcpA+CTB or TcpA+FliC (Fig. 6A, P>0.05). Similarly, the mean concentrations of serum anti-CTB IgG at day 42 did not differ significantly in dams immunized with TcpA-A2-CTB chimera, TcpA+CTB, or CTB alone (Fig. 5B), and the mean percentages of fecal anti-CTB IgA did not differ significantly among dams in these immunization groups (Fig. 6B). Published studies show that transfer of maternal antibodies to pups (which can occur in utero, by suckling, or by both pathways) is the primary mechanism by which immunization of dams confers immunologically specific protection to their pups [28]–[30]. Therefore, the complete protection achieved in pups from dams immunized with the TcpA-A2-CTB chimera or the TcpA+CTB mixture, versus the lack of any protection in pups from dams immunized with TcpA+FliC or CTB alone, cannot be explained either by poorer serum or fecal anti-TcpA or anti-CTB antibody responses, respectively, vs. the comparable antigen-specific antibody responses in the mice immunized with the TcpA-A2-CTB chimera or the TcpA+CTB mixture.

### Analysis of weight loss in the suckling mouse model of cholera

**Figure 8:**
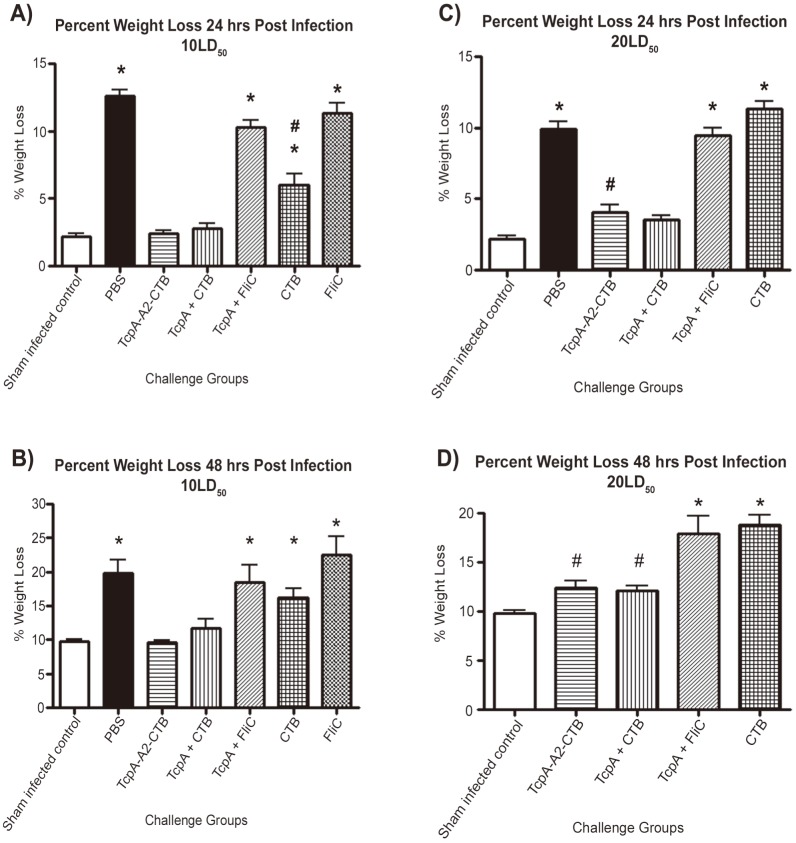
Average weight losses of 6-day old infant mice born to immunized or non-immunized dams at 24 and 48 hrs following challenge with a 10 or 20 LD_50_ dose of *V. cholerae* El Tor strain N16961. Living mouse pups in each immunization group were weighed immediately prior to and at 24 and 48 hrs after challenge with *V. cholerae*. For pups that died before 24 hrs or between 24 and 48 hrs, their carcasses were weighed at 24 hrs or 48 hrs, respectively, and the corresponding weights were included in the data presented for 24 and 48 hrs. Weight losses at 24 hrs (A) and 48 hrs (B) after challenge with 10 LD_50_ of *V. cholerae*. Weight losses at 24 hrs (C) and 48 hrs (D) after challenge with 20 LD_50_ of *V. cholerae*. (Note: data for weight loss at 48 hrs in the PBS-immunized control group is not shown in panel D because all mice in this control group were dead by 24 hrs). Statistical differences between groups were performed using ANOVA with the Tukey-Kramer post-test. In 8A, * indicates P<0.001 compared to values for the sham-infected control group and the TcpA-A2-CTB and TcpA+CTB immunized groups; and # indicates P<0.001 compared to values for PBS, TcpA+FliC, and FliC immunized groups. In 8B, * indicates P<0.05 compared to values for the sham infected control group and the TcpA-A2-CTB and TcpA+CTB immunized groups. In 8C, * indicates P<0.001 compared to values for the sham infected control group and the TcpA-A2-CTB and TcpA+CTB immunized groups; and # indicates P<0.05 compared to values for the sham-infected control group. In 8D, * indicates P<0.001 compared to values for the sham-infected control group and the TcpA-A2-CTB and TcpA+CTB immunized groups; and # indicates P<0.05 compared to values for the sham-infected control group.

Sham-infected pups experienced about 2% weight loss at 24 hrs and 10% weight loss at 48 hrs because they were separated from their dams since 3 hrs before challenge (Fig. 8). In addition, mouse pups develop diarrhea if they are not fully protected against *V. cholerae* infection by active or passive immunity [31]. At the 10 LD_50_ challenge dose of *V. cholerae* N16961, pups from dams immunized with the TcpA-A2-CTB chimera or TcpA+CTB mixture did not lose significantly more weight at 24 or 48 hrs than sham-infected pups (Figs. 8A and 8B; P>0.05), and all survived for 48 hrs (Fig. 7A). In contrast, pups from PBS immunized dams or dams immunized with TcpA+FliC or FliC alone did experience much greater weight losses than sham-infected pups both at 24 hrs (Fig. 8A; P<0.001) and at 48 hrs (Fig. 8B; P<0.05), and all died by 48 hrs (Figs. 7A and 7B). The severity and timing of their excess weight losses and their death within 48 hrs reflected the onset of severe diarrhea. Pups from dams immunized with CTB experienced less-dramatic excess weight losses at 24 hours than pups from dams immunized with PBS, TcpA+FliC, or FliC (Fig. 8A; P<0.001), but they experienced greater weight losses than sham-infected pups at 24 and 48 hrs (Figs. 8A and 8B; P<0.001). This resulted in a 70% survival rate of CTB immunized pups at 48 hrs. These findings indicate that pups from dams immunized with CTB were partially protected against challenge with 10 LD_50_ of *V. cholerae* N16961, and they experienced less severe diarrhea than PBS immunized pups (Figs. 7A and 7B).

At the 20 LD_50_ challenge dose, pups from dams immunized with the TcpA-A2-CTB chimera exhibited significantly greater weight losses at 24 hrs than sham-infected control pups (Fig. 8C; *P*<0.05), and pups from dams immunized either with the TcpA-A2-CTB chimera or with TcpA+CTB exhibited significantly greater weight losses at 48 hrs than sham-infected control pups (Fig. 8D, P<0.05) although all pups in both groups survived for 48 hrs. The excess weight losses among pups in these two groups indicate that they experienced mild diarrhea at the 20 LD_50_ challenge dose. The challenged pups from dams immunized with TcpA+FliC or CTB alone experienced much greater weight losses than the sham-infected controls (P<0.001; Figs. 8C+8D), and all of them died before 48 hrs, indicating that they experienced severe diarrhea at the 20 LD_50_ challenge dose.

### Measurements of anti-TcpA and anti-CTB IgG antibody concentrations in the serum of six-day old infant mice from immunized dams

**Figure 9:**
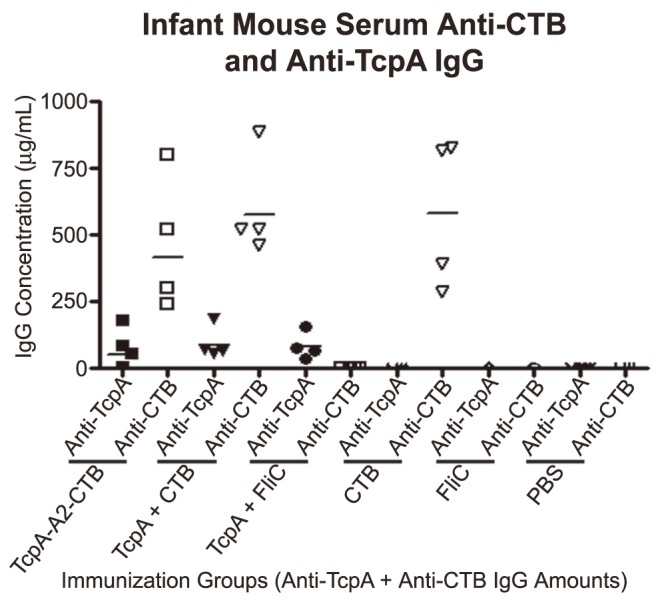
Concentrations of anti-TcpA and anti-CTB IgG antibodies in sera collected from 6-day old pups born to immunized dams. One or two pups per dam from the groups immunized with the TcpA-A2-CTB chimera or with TcpA+CTB, TcpA+FliC, CTB, FliC or PBS were sacrificed, on the same day that their siblings were challenged with *V. cholerae*, to obtain serum for measurements of anti-TcpA and anti-CTB IgG antibody concentrations. Each data point is the serum anti-TcpA or anti-CTB IgG antibody concentration representing the litter of pups from one immunized dam used in the challenge studies with *V. cholerae*. Filled symbols represent anti-TcpA IgG concentrations, and open symbols represent anti-CTB IgG concentrations. Horizontal lines represent the geometric mean IgG concentration of anti-TcpA or anti-CTB IgG antibodies for all litters of pups born to dams in the indicated immunization group.

One or two pups from each dam in the immunization groups included in the suckling mouse challenge studies described above were sacrificed at the same time that their siblings were challenged to obtain serum samples for measurements of antigen-specific IgG concentrations by quantitative ELISA. As shown in Fig. 9, the mean anti-TcpA IgG serum antibody concentrations from the pups were statistically equivalent regardless of whether their dams were immunized with TcpA-A2-CTB chimera, TcpA+CTB, or Tcp+FliC (*P*>0.05). The mean anti-CTB IgG serum antibody concentrations from the pups were also statistically equivalent regardless of whether their dams were immunized with TcpA-A2-CTB chimera, TcpA+CTB, or CTB (*P*>0.05). Consistent with results shown previously for serum antibodies at day 42 in immunized dams, the mean concentrations of antigenic-specific serum IgG antibodies from these pups were much greater for the anti-CTB antibodies than for anti-TcpA antibodies. None of the sera from pups had detectable anti-TcpA-specific IgA antibodies, and only one serum from a pup born to a dam immunized with TcpA-A2-CTB chimera had detectable anti-CTB-specific IgA antibodies.

## Discussion

Early studies showed that pups from non-immunized dams survived large orogastric challenge doses of *V. cholerae* (500–2000 LD_50_) when the bacteria were pre-mixed with anti-CT or anti-TCP antiserum [23], [32], but pups from dams immunized against TcpA or CTB survived only when challenge doses were much smaller (1–15 LD_50_) [21], [26], [33]. Titers of serum anti-TcpA IgG1 and IgA antibodies in dams correlated with survival rates of their challenged pups [33]. Survival rates of challenged pups from immunized dams fell more rapidly as the dam's log_10_ anti-TcpA IgG1 titers decreased than did survival rates of pups from unimmunized dams with comparable decreases in anti-TCP antiserum doses [33]. Pups given anti-TCP antiserum intraperitoneally also survived *V. cholerae* challenges given 24 hrs later [23]. Taken together, these findings show that intestinal anti-TcpA or anti-CTB antibodies protect infant mice from potentially lethal *V. cholerae* challenges and indicate that maternal antibodies are delivered into the intestines of infant mice either actively by suckling or passively by transudation from internal body fluids.

In the studies reported here, we investigated whether immunizing dams with TcpA-A2-CTB chimera or TcpA+CTB protected their pups more effectively than immunizing dams with TcpA or CTB alone in the infant mouse model of cholera. We challenged separate groups of pups with 10 LD_50_ and 20 LD_50_ doses of *V. cholerae* El Tor N16961 to assess protection under stringent conditions.

Few previous studies compared protective efficacy of immunization with TcpA+CT (or CTB) vs. TcpA or CT (or CTB) alone in animal models of cholera. Transcutaneous immunization (TCI) of dams with CT+TcpA protected pups better against a 1 LD_50_ challenge with *V. cholerae* N16961 (69% survival) than did TCI with CT alone (36% survival), but TCI with TcpA alone induced no detectable anti-TcpA antibodies in dams and their pups were not challenged [25]. Although that study and our study used different methods to measure antigen-specific antibodies, the more robust protection of pups at higher challenge doses that we observed likely indicates higher serum anti-TcpA and anti-CTB antibody levels in our immunized dams. Other investigators used the ligated ileal segment model in adult rabbits to compare protection conferred by intranasal immunization with TcpA+CTB, TcpA alone, or CTB alone [34]. Fluid accumulation in ligated ileal segments decreased by 41.1% vs. unimmunized controls in rabbits immunized with TcpA and by 70.5% in rabbits immunized with CTB, but no fluid accumulated in ligated ileal segments of rabbits immunized with TcpA+CTB [34]. The immunized rabbits also developed intestinal sIgA antibodies against the TcpA and/or CTB antigens that they received [34].

In our studies (see Fig. 7), all pups from dams immunized with TcpA-A2-CTB chimera or TcpA+CTB survived 48 hrs after a 10 or 20 LD_50_ challenge dose of *V. cholerae* El Tor N16961; no pups from dams immunized with TcpA+FliC survived 48 hrs at either challenge dose; and pups from dams immunized with CTB had 70% 48-hr survival at the 10 LD_50_ challenge dose a 0% 48-hr survival at the 20 LD_50_ dose. At each challenge dose, the 100% survival rate for pups with both anti-TcpA and anti-CTB antibodies significantly exceeded the sum of the survival rates for pups with only anti-TcpA antibodies and for pups with only anti-CTB antibodies [*e.g.*, (0%+70%) = 70% cumulative survival at 10 LD_50_ and (0%+0%) = 0% cumulative survival at 20 LD_50_ among pups with only anti-TcpA antibodies plus pups with only anti-CTB antibodies]. Because these differences in survival could not be explained by significant differences in mean values of TcpA-specific or CTB-specific serum IgG or fecal IgA antibodies among groups of pups from dams immunized with vaccine formulations that contained any form of TcpA or CTB, respectively (see Figs. 5 and 6), our results constitute strong preliminary evidence that anti-TcpA and anti-CTB antibodies act synergistically rather than additively to prevent death in the infant mouse model of cholera.

In humans, cholera is caused either by *V. cholerae* serogroup O1 (with classical and El Tor biotypes and Inaba and Ogawa serotypes) or *V. cholerae* serogroup O139 (first recognized in 1992–1993) [2], [3]. Early clinical isolates of *V. cholerae* O139 were closely related to *V. cholerae* O1 El Tor (but with different genes at the O antigen locus), but later *V. cholerae* O139 isolates belong to multiple lineages derived from different *V. cholerae* progenitors [35], [36]. The O1 and O139 lipopolysaccharides are essential for virulence of *V. cholerae* O1 and O139 in humans, are important protective antigens, and elicit serogroup-specific antibodies that do not cross-react with each other [36]–[38]. CT and TcpA are also essential for virulence of *V. cholerae* O1 and O139 and are immunogenic in humans [16], [17], [39]–[41], but analyzing their roles in protective immunity is complicated by the existence of multiple antigenically cross-reacting variants of each protein among classical, El Tor and “hybrid El Tor” isolates of *V. cholerae* O1 and *V. cholerae* O139 [22], [23], [26], [42]–[44].

Early studies in human volunteers suggested that immunity against cholera was mediated primarily by antibacterial rather than antitoxic mechanisms [20], and for decades the best serological (but non-mechanistic) correlate of protection among patients and volunteers who recovered from a previous episode of cholera was the titer of complement-dependent vibriocidal antibodies [45]. More recent studies in humans showed that serum IgA (but not IgG) antibodies against CTB, LPS, or TcpA also correlate with protection against cholera [3], [46], [47]. However, because recovery confers protection against a future episode of cholera that persists much longer than titers of vibriocidal, anti-CTB, anti-LPS, or anti-TcpA antibodies remain elevated, prompt anamnestic antibody responses following exposure to *V. cholerae* are believed to be important for long-term immunity against cholera. Consistent with this view, patients who recover from cholera have been found to develop IgG and IgA memory B (B_M_) cells specific for LPS, TcpA, and CTB as well as effector memory T (TEM) cells specific for CTB [45]–[48].

The mechanisms by which intestinal anti-CTB and anti-TcpA antibodies protect against cholera (e.g., blocking CT-mediated toxicity and TCP-mediated contributions to colonization) are believed to be similar in humans and in infant mice. Our results provide proof-of-principle that immunizing dams with TcpA-A2-CTB chimera or TcpA+CTB can protect 100% of pups against challenges with up to 20 LD_50_ of *V. cholerae* El Tor N16961, which produces CTB and TcpA variants homologous to those used for immunization. To the best of our knowledge, no other reported immunization regimen for dams protects pups so well against such a stringent challenge in the infant mouse model of cholera. Further studies will be needed: 1) to assess the relative protective efficacy of current TcpA-A2-CTB and TcpA+CTB vaccines against challenges with *V. cholerae* O1 classical or El Tor or *V. cholerae* O139 strains that produce homologous or heterologous variants of CTB and TcpA; and 2) to determine whether immunization with at least two variants each of TcpA and CTB can provide broader protection than immunization with one variant of each protein against *V. cholerae* challenge strains that produce several different CTB and/or TcpA variants. Using a TcpA-A2-CTB chimera instead of a TcpA+CTB mixture in a vaccine formulation has several potential advantages, since the chimera is a chemically defined, highly immunogenic, macromolecular complex that can be assembled spontaneously in *E. coli* and purified as a single entity. If necessary, different variants of TcpA and CTB could be incorporated into different chimeras, which could then be combined to create a vaccine formulation containing multiple variants of CTB and TcpA.

The WCK oral cholera vaccine that provided significant protection for 5 years in an endemic region does not contain CTB, and it is unclear whether the heat and formalin treatments used to inactivate *V. cholerae* during preparation of that vaccine cause any damage to the immunogenicity of TcpA or other protein protective antigens of the bacteria [5], [6]. Our results show clearly that TcpA and CTB can be used successfully as protective subunit immunogens against cholera in the infant mouse model. Extending to humans the potential value of incorporating TcpA and CTB into effective vaccines against cholera will require additional studies to address the need: 1) to elicit production of antigen-specific sIgA antibodies in the human intestine; 2) to achieve long-term memory for protective intestinal immune responses; and 3) to develop vaccine formulations, adjuvants, routes of delivery, and immunization regimens to accomplish these goals.
